# Clinical Characteristics, Prognosis, and Nomogram for Esophageal Cancer Based on Adenosquamous Carcinoma: A SEER Database Analysis

**DOI:** 10.3389/fonc.2021.603349

**Published:** 2021-04-26

**Authors:** Haisheng Qian, Xiaofeng Ji, Chang Liu, Yini Dang, Xuan Li, Guoxin Zhang

**Affiliations:** ^1^Department of Gastroenterology, The First Affiliated Hospital of Nanjing Medical University, Nanjing, China; ^2^Department of Gastroenterology, The First School of Clinical Medicine of Nanjing Medical University, Nanjing, China; ^3^School of Pediatrics, Nanjing Medical University, Nanjing, China

**Keywords:** esophageal cancer, adenosquamous carcinoma, adenocarcinoma, squamous cell carcinoma, surveillance, epidemiology and end results (SEER) database, nomogram

## Abstract

**Objective:** Esophageal adenosquamous carcinoma (ASC) is a rare pathological type of cancer. Its clinical features and prognosis is poorly understood. The purpose of this study was to identify the characteristics of ASC patients and analyze the risk factors of esophageal carcinoma.

**Methods:** Patients with esophageal cancer in the SEER database diagnosed from 1975–2016 were obtained. The epidemiology, clinical characteristics, and outcomes between these three groups were compared. The nomogram and online dynamic nomogram were constructed according to the Cox proportional hazard model.

**Results:** The age-adjusted incidences of AC (1975–1999), AC (1999–2016), and ASC (1975–1989) increased over time (*p* < 0.05). Age-adjusted incidences of SqCC (1986–2012) and ASC (1989–2016) decreased (*p* < 0.05). Survival of patients with ASC was significantly worse when compared to AC and SqCC (ASC vs. AC, *p* < 0.001, ASC vs. SqCC, *p* = 0.01). ASC, older age, black race, male, overlapping site, higher tumor grade, lymph node metastasis, and a higher summary stage or AJCC stage were considered to be risk factors for a poor survival in the multivariate Cox analysis. The ROC curves and AUC indicated that the model has a good discrimination ability (AUC were 0.774 for a 3-year OS and 0.782 for a 5-year OS). An online dynamic nomogram was built based on the Cox proportional hazard model for convenient clinical use.

**Conclusions:** ASC is somewhat closer to AC rather than SqCC in terms of the demographics and tumor site, but has a worse OS than both AC and SqCC.

## Introduction

Esophageal cancer is the eighth most common cancer worldwide and the sixth leading cause of cancer-related mortality worldwide (1, 2). The two major histological types of esophageal cancer are Adenocarcinoma (AC) and Squamous Cell Carcinoma (SqCC), in which the latter is the most prevalent esophageal cancer worldwide (1). Adenosquamous carcinoma (ASC) of the esophagus is a rare type of esophageal cancer, which is comprised of both AC and SqCC elements (3). Due to its rare incidence, ASC of the esophagus was rarely reported, most of which were single case reports or small case series (4–12). These studies provided contradictory information regarding the clinical characteristics and prognosis of ASC. It was also unknown that its biological behaviors were generally determined by the AC or SqCC component. No consensus regarding these questions about ASC has been formed, so more studies are needed to explore the pathogenesis, biological behavior, treatment, clinical characteristics, and prognosis of ASC, and the differences among ASC, AC, and SqCC.

The Surveillance, Epidemiology, and End Results (SEER) Program of the National Cancer Institute collects and publishes cancer incidence and survival data from population-based cancer registries covering ~34.6% of the United States. It is a useful resource to study rare tumors like ASC. ASC is somewhat closer to AC rather than SqCC in terms of demographics and tumor site, but has a worse OS than both AC and SqCC. Therefore, our study was conducted to explore the epidemiology, clinical characteristics, and prognosis among patients with ASC, AC, and SqCC *via* the SEER database. In addition, we also analyzed the risk factors of esophageal carcinoma and constructed a nomogram to help predict outcomes in clinical work.

## Methods

### Patient Selection

To describe the incidence of esophageal ASC, AC, and SqCC, we first extracted cases of esophageal carcinoma diagnosed during 1975 and 2016 from the SEER database (SEER^*^Stat 8.3.6) according to the site recode classifications. Considering that the American Joint Committee on Cancer (AJCC) staging manual (7th edition) was available since 2010, cases of esophageal carcinoma diagnosed from 2010 to 2016 were selected Histological type limited to adenocarcinoma (ICD-03, 8140–8144, 8210, 8211, 8255, 8260–8263, 8310, 8480, 8481, 8570, 8574, 8576), squamous cell carcinoma (ICD-03, 8052, 8053, 8070–8078, 8083, 8084, 8094), and Adenosquamous carcinoma (ICD-03, 8560). Clinical characteristics were extracted from the database including: age, sex, race, grade, summary stage, AJCC Stage, T stage, lymph node metastasis, distant metastasis, and follow-up vital status.

### Statistical Analysis

Age-adjusted incidence as diagnoses per 100,000 patients per year was calculated using the SEER^*^Stat (Version 8.3.6). Annual percentage changes (APCs) for assessing the changes of incidence were estimated by the Joinpoint software (version 4.7.0). The intergroup comparison of clinicopathologic variables was performed using the chi-square test for categorical covariates and analysis of variance for numerical covariates. Survival was analyzed using the Kaplan-Meier method and log-rank test. We generated the 1:1 matched ASC/AC group and ASC/SqCC group *via* a propensity score matching (PSM) method to reduce the effects of differences in baseline features. Age at diagnosis, gender, race, pathological grade, summary stage, AJCC Stage, T stage, lymph node metastasis, distant metastasis, and primary site were included for matching. Cox proportional regression analysis was performed for the univariate and multivariate analysis of prognostic factors, including: pathological type, age at diagnosis, gender, race, grade, summary stage, AJCC Stage, T stage, lymph node metastasis, distant metastasis, and primary site. The nomogram was constructed according to the result of the univariate and multivariate analysis using the “rms” package. The nomogram model validation was performed by AUC and C-index for discrimination ability, and calibration curves for calibration ability. Bootstraps with 1,000 resamples were adopted to decrease the overfit bias. The *p*-value < 0.05 was considered to be significant. Statistical analysis was performed with the SPSS 22.0 and R software version 3.6.2.

## Results

### Incidence of Three Histological Types

**Figure 1:**
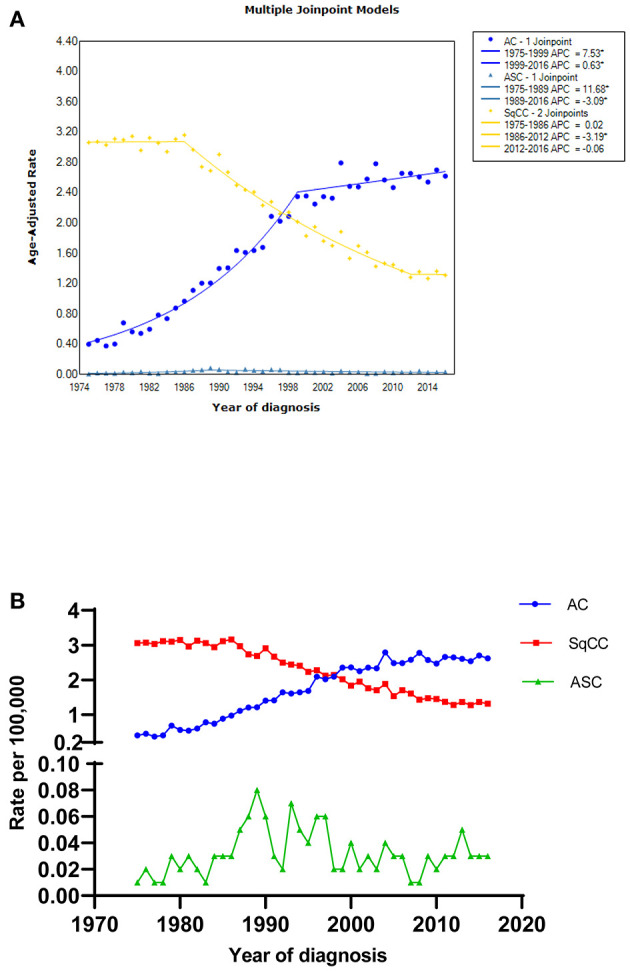
The age-adjusted incidence of esophageal AC, SqCC, and ASC patients between 1975 and 2016 from the SEER database. **(A)** Figure drawn by the Joinpoint software; **(B)** Figure drawn by the GraphPad Prism 6 software; * indicated that the APC is significantly different from zero at the alpha = 0.05 level. AC, adenocarcinoma; SqCC, squamous cell carcinoma; ASC, adenosquamous carcinoma; APC, annual percent change.

To describe the incidence of these three histological types, we selected esophageal ASC, AC, and SqCC patients during 1975 and 2016 from the SEER database. The results showed that the age-adjusted incidences of AC (1975–1999), AC (1999–2016), and ASC (1975–1989) significantly increased over time (*p* < 0.05), with the APCs of 7.5% (95% CI: 6.9–8.2), 0.6% (95% CI: 0.1–1.2), and 11.7% (95% CI: 4.8–19), respectively. In contrast, age-adjusted incidences of SqCC (1986–2012) and ASC (1989–2016) significantly decreased (*p* < 0.05), with the APCs of −3.2% (95% CI: −3.4 to −3) and −3.1% (95% CI: −4.6 to −1.5). Furthermore, there was no distinct increase nor decrease in the trend for the incidence of SqCC (1975–1986) and SqCC (2012–2016) (Figure 1 and Supplementary Table 1).

### Demographic and Clinical Characteristics

A total of 20,027 esophageal cancer patients from 2010 to 2016 including 13,248 AC cases, 6,627 SqCC cases, and 152 ASC cases were identified through the SEER database. Table 1 summarized the clinical characteristics of these three histologic subgroups. The mean age at diagnosis was 67.2 for AC, 68.1 for SqCC, and 65.1 for ASC (*p* < 0.001).

**Table 1:** Characteristics of patients with esophageal ASC, AC, and SqCC.

**Characteristics**	**AC**	**ASC**	**SqCC**	**Total**	***P*-value**	**ASC vs. AC**	**ASC vs. SqCC**	**AC vs. SqCC**
Age, years/SEM	67.2/11.4	65.7/11.7	68.1/11.1	67.5/11.3	<0.001	0.103	0.09	<0.001
Gender, *n* (Col %)					<0.001	0.044	<0.001	<0.001
Male	11,464 (86.5)	123 (80.9)	4,283 (64.6)	15,870 (79.2)				
Female	1,784 (13.5)	29 (19.1)	2,344 (35.4)	4,157 (20.8)				
Race, *n* (Col %)					<0.001	0.049	<0.001	<0.001
White	12,539 (94.6)	137 (90.1)	4,296 (64.8)	16,972 (84.7)				
Black	366 (2.8)	8 (5.3)	1,609 (24.3)	1,983 (9.9)				
Other	343 (2.6)	7 (4.6)	722 (10.9)	1,072 (5.4)				
Pathological grade, *n* (Col %)					<0.001	<0.001	<0.001	<0.001
Grade I	716 (5.4)	0 (0)	323 (4.9)	1,039 (5.2)				
Grade II	4,443 (33.5)	20 (13.2)	2,687 (40.5)	7,150 (35.7)				
Grade III	5,582 (42.1)	100 (65.8)	2,242 (33.8)	7,924 (39.6)				
Grade IV	141 (1.1)	2 (1.3)	36 (0.5)	179 (0.9)				
Unknown	2,366 (17.9)	30 (19.7)	1,339 (20.2)	3,735 (18.6)				
Summary stage, *n* (Col %)					<0.001	<0.001	<0.001	<0.001
Localized	2,888 (21.8)	16 (10.5)	1,335 (20.1)	4,239 (21.2)				
Regional	4,675 (35.3)	48 (31.6)	2,806 (42.3)	7,529 (37.6)				
Distant	5,685 (42.9)	88 (57.9)	2,486 (37.5)	8,259 (41.2)				
AJCC stage, *n* (Col %)					<0.001	0.002	<0.001	<0.001
I	2,651 (20)	13 (8.6)	1,055 (15.9)	3,719 (18.6)				
II	2,149 (16.2)	22 (14.5)	1,523 (23)	3,694 (18.4)				
III	3,538 (26.7)	44 (28.9)	2,208 (33.3)	5,790 (28.9)				
IV	4,910 (37.1)	73 (48)	1,841 (27.8)	6,824 (34.1)				
T Stage, *n* (Col %)					<0.001	0.042	0.226	<0.001
T1	3,639 (27.5)	30 (19.7)	1,689 (25.5)	5,358 (26.8)				
T2	1,456 (11)	14 (9.2)	747 (11.3)	2,217 (11.1)				
T3	4,852 (36.6)	57 (37.5)	2,336 (35.2)	7,245 (36.2)				
T4	3,301 (24.9)	51 (33.6)	1,855 (28)	5,207 (26)				
LN metastasis, *n* (Col %)					0.003	0.002	0.001	0.162
No	5,283 (39.9)	42 (27.6)	2,711 (40.9)	8,036 (40.1)				
Yes	7,965 (60.1)	110 (72.4)	3,916 (59.1)	11,991 (59.9)				
M stage, *n* (Col %)					<0.001	0.005	<0.001	<0.001
M0	8,338 (62.9)	79 (52)	4,786 (72.2)	13,203 (65.9)				
M1	4,910 (37.1)	73 (48)	1,841 (27.8)	6,824 (34.1)				
Primary site, *n* (Col %)					<0.001	<0.001	<0.001	<0.001
Cervical/upper	152 (1.1)	3 (2)	1,425 (21.5)	15,80 (7.9)				
Thoracic/middle	1,130 (8.5)	39 (25.7)	2,898 (43.7)	4,067 (20.3)				
Abdominal/lower	11,400 (86.1)	95 (62.5)	1,947 (29.4)	13,442 (67.1)				
Overlapping	566 (4.3)	15 (9.9)	357 (5.4)	938 (4.7)				

*AC, adenocarcinoma; SqCC, squamous cell carcinoma; ASC, adenosquamous carcinoma; LN, lymph node*.

In terms of the demographic features, ASC seems closer to AC than SqCC. The mean age at diagnosis was 67.2 for AC, 65.7 for ASC, and 68.1 for SqCC. With regard to gender, all three histological groups showed a higher burden of disease in men, with males comprising 86.5% of AC, 80.9% of SqCC, and 64.6% of ASC cases (*p* < 0.001). For race, all three types were most prevalent in whites, but SqCC exhibited a significantly greater proportion in blacks with 24.3% of cases vs. 2.8% of ASC and 5.3% of AC (*p* < 0.001). When it comes to the clinical features, ASC tumors tended to present a later summary stage or AJCC stage, a later T stage, a higher lymph node metastasis rate, and a higher distant metastasis.

### Survival Analyses

#### Kaplan-Meier Survival Curves of All Three Histological Types Patients

**Figure 2:**
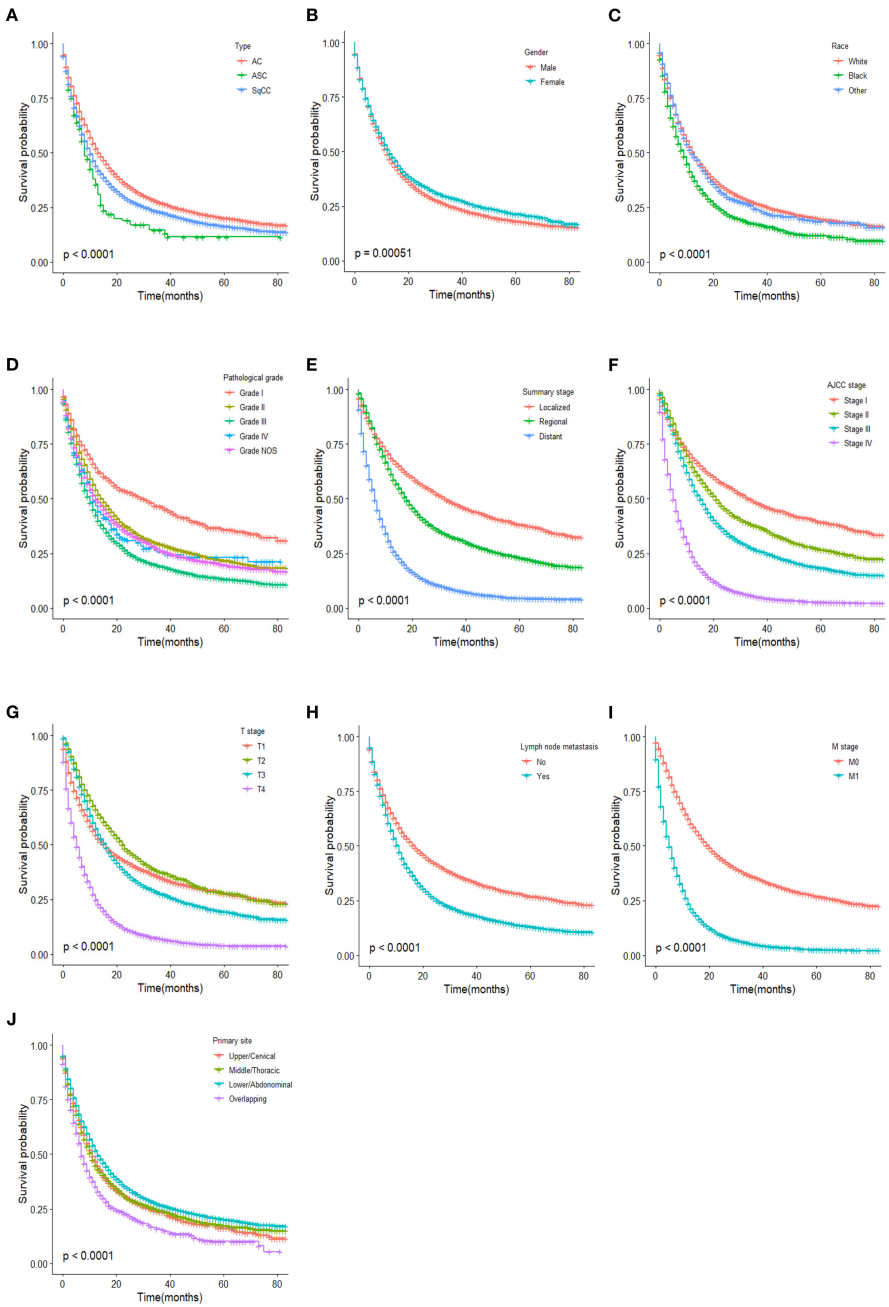
Kaplan–Meier estimated overall survival in patients stratified by type **(A)**, gender **(B)**, race **(C)**, pathological grade **(D)**, summary stage **(E)**, AJCC stage **(F)**, T stage **(G)**, lymph node metastasis **(H)**, M stage **(I)**, primary site **(J)**.

We used the Kaplan-Meier method to evaluate the overall survival among all three types of patients (Figure 2). As shown in Figure 2A, survival of patients with ASC was significantly worse when compared to AC and SqCC, and patients with AC showed a better OS than the other two types (ASC vs. AC, *p* < 0.001, ASC vs. SqCC, *p* = 0.01, AC vs. SqCC, *p* < 0.001). In terms of gender and race, the male and black patients had a shorter OS than patients who were female and had other races (*p* < 0.001, respectively, Figures 2B,C). Interestingly, pathological grade III was significantly correlated to a worse OS than Grade IV (*p* = 0.036, Figure 2D). Figure 2J exhibited that the overlapping site of cancer had a worse OS than other sites (*p* < 0.001).

To further explore the relationship between the tumor type and OS, we drew the Kaplan-Meier survival curves of the three histological types stratified by gender, race, pathological grade, summary stage, AJCC stage, T stage, lymph node metastasis, M stage, and primary site (Supplementary Figure 1). As the log-rank test results of the three histological types in different stratification variables are shown in Supplementary Table 2, the OS between ASC and SqCC patients had a statistical difference only in the white race and pathological Grade NOS stratification variables (*p* = 0.006 and 0.007, respectively). However, the statistical difference of the OS between ASC and AC patients was found in male, white race, Grade NOS, localized tumor stage, AJCC stage I, AJCC stage III, T1 stage, T4 stage, lymph node metastasis, and M0 stage. In most stratification variables, OS of SqCC patients was statistically different from AC patients. For patients after PSM, there was no statistical difference in the OS of esophageal ASC compared to AC and SqCC (Supplementary Figure 2, ASC vs. AC, *p* < 0.59, ASC vs. SqCC, *p* = 0.94).

#### Kaplan-Meier Survival Curves of ASC Patients

**Figure 3:**
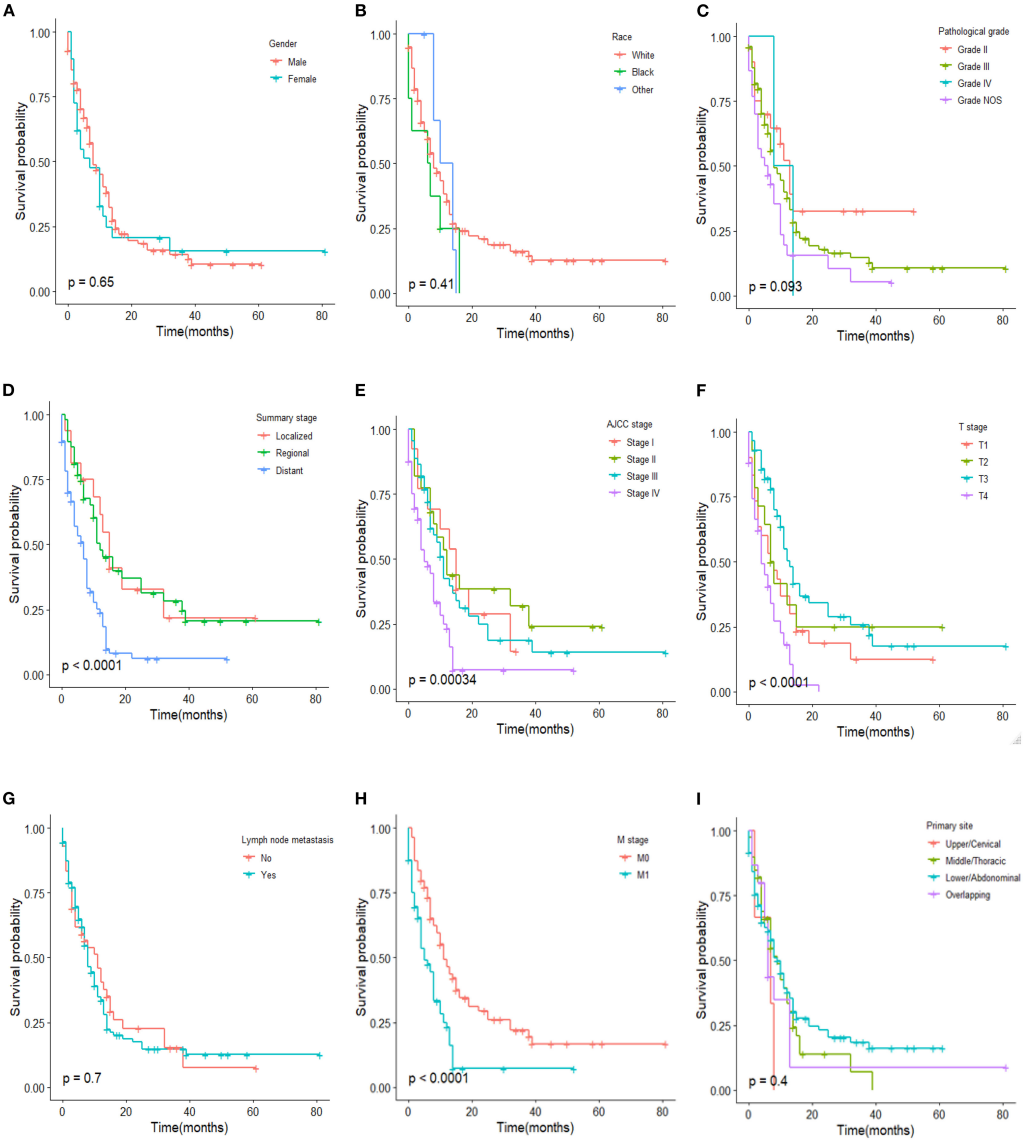
Kaplan–Meier estimated overall survival in ASC patients stratified by gender **(A)**, race **(B)**, pathological grade **(C)**, summary stage **(D)**, AJCC stage **(E)**, T stage **(F)**, lymph node metastasis **(G)**, M stage **(H)**, primary site **(I)**.

Kaplan-Meier (KM) survival curves of ASC patients are shown in Figure 3. In contrast to the results above, there was no statistical difference in ASC patients between the male and female or among different races (*p* = 0.65 and 0.41, respectively). It is interesting that lymph node metastasis had no influence on the OS of ASC patients (*p* = 0.700). Also, for the summary stage, AJCC stage, and T stage, no difference of OS in ASC patients was found between the localized and regional stages (*p* = 0.954), among AJCC stages I–III (*p* = 0.681), and among T1–3 (*p* = 0.120).

### Predictors of Mortality

Univariate and multivariate Cox analysis was performed to identify the prognostic factors associated with the OS in three histological types of patients (Table 2). Univariate analysis and multivariate Cox showed that ASC and SqCC histological type, older age, black race, male, overlapping site, higher tumor grade, lymph node metastasis, higher summary stage, and AJCC stage were recognized as significant risk factors for a poor survival. M stage was excluded from the multivariate Cox analysis because of its strong correlation with AJCC stage IV.

**Table 2:** Univariate and multivariate cox analysis of clinical characteristics for the OS in all three esophageal cancer type patients.

**Variable**	**Reference**	**Univariate**		**Multivariate**	
		**HR (95%CI)**	***P*-value**	**HR (95%CI)**	***P*-value**
Age (>65)	≤ 65	1.18 (1.14–1.22)	<0.001	1.33 (1.28–1.37)	<0.001
Type	AC				
ASC		1.53 (1.27–1.83)	<0.001	1.25 (1.04–1.5)	0.016
SqCC		1.19 (1.15–1.23)	<0.001	1.18 (1.12–1.24)	<0.001
Gender (female)	Male	0.93 (0.89–0.97)	0.001	0.93 (0.89–0.97)	0.001
Race	White				
Black		1.36 (1.29–1.44)	<0.001	1.22 (1.15–1.29)	<0.001
Other		1.03 (0.96–1.11)	0.408	0.93 (0.86–1.01)	0.079
Pathological grade	Grade I				
Grade II		1.48 (1.35–1.62)	<0.001	1.25 (1.14–1.37)	<0.001
Grade III		2.03 (1.86–2.21)	<0.001	1.53 (1.4–1.67)	<0.001
Grade IV		1.66 (1.36–2.02)	<0.001	1.3 (1.07–1.59)	0.008
Grade NOS		1.64 (1.49–1.8)	<0.001	1.32 (1.2–1.45)	<0.001
Summary stage	Localized				
Distant		3.45 (3.28–3.63)	<0.001	1.58 (1.37–1.83)	<0.001
Regional		1.42 (1.35–1.5)	<0.001	1.28 (1.12–1.45)	<0.001
AJCC stage	Stage I				
Stage II		1.29 (1.21–1.37)	<0.001	1.23 (1.08–1.41)	0.002
Stage III		1.68 (1.59–1.78)	<0.001	1.6 (1.38–1.85)	<0.001
Stage IV		4.15 (3.93–4.38)	<0.001	2.77 (2.38–3.23)	<0.001
T stage	T1				
T2		0.82 (0.76–0.87)	<0.001	0.78 (0.72–0.84)	<0.001
T3		1.06 (1.01–1.1)	0.017	0.76 (0.72–0.81)	<0.001
T4		2.56 (2.44–2.68)	<0.001	1.19 (1.12–1.26)	<0.001
LNM (yes)	No	1.46 (1.41–1.51)	<0.001	1.17 (1.12–1.13)	<0.001
M stage (M1)	M0	3.06 (2.95–3.17)	<0.001		
Primary site	Cervical/upper				
Middle/thoracic		0.99 (0.92–1.06)	0.8	1.01 (0.94–1.09)	0.728
Lower/abdominal		0.88 (0.82–0.93)	<0.001	0.95 (0.88–1.02)	0.163
Overlapping		1.34 (1.22–1.47)	<0.001	1.21 (1.09–1.33)	<0.001

*AC, adenocarcinoma; SqCC, squamous cell carcinoma; ASC, adenosquamous carcinoma; LNM, lymph node metastasis; OS, overall survival*.

**Figure 4:**
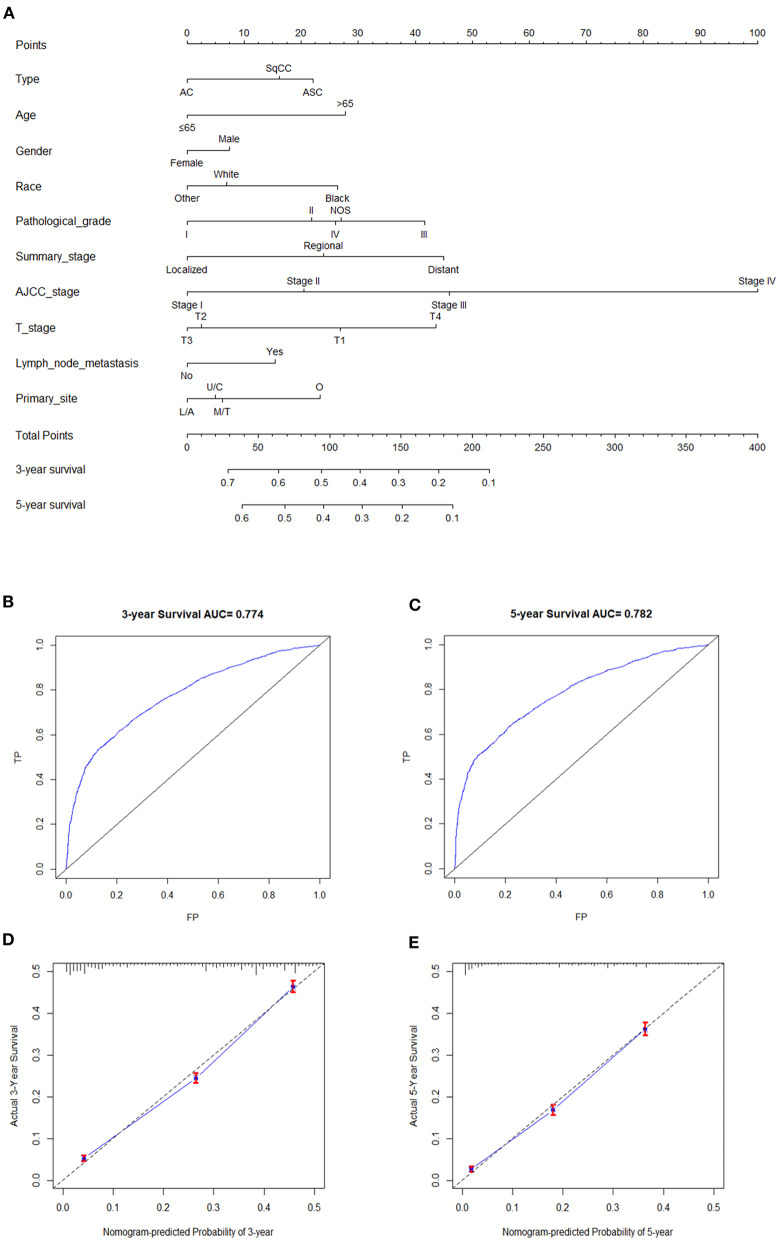
**(A)** Nomogram for all three cancer types patients. C/U, Cervical/Upper; T/M, Thoracic/Middle; A/L, Abdominal/Lower; O, Overlapping. **(B,C)** are ROC curves and AUC of nomogram model for 3- and 5-year OS. **(D,E)** are calibration curves of 3- and 5-year OS rates.

The nomogram was constructed according to the Cox proportional hazard model. An online version of the nomogram was available at https://qhschn-tools.shinyapps.io/Nomogram-for-EsophagealCancer/ for convenient clinical use and future validation. Histological type, age, gender, race, pathological grade, summary stage, AJCC stage, T stage, lymph node metastasis, and primary site were included. The prediction results of the 3- and 5-year OS rates are shown in Figure 4A. The ROC curves and AUC indicated that the model has a good discrimination ability (Figures 4B,C, AUC were 0.774 for a 3-year OS and 0.782 for a 5-year OS). Additionally, the calibration curves of the 3- and 5-year OS rates showed the model fits well (Figures 4D,E).

## Discussion

Consistent with the existing literature, the incidence of esophageal ASC is extremely low, only comprising <1% of all esophageal malignancies (ASC: 0.76%, AC: 66.15%, 33.09%). It is worth noting that the incidence of ASC from 1989 to 2016 and SqCC from 1986–2012 decreased over time in the United States, which is likely attributed to changes in smoking and diet habits. However, there was an increasing trend for the incidence of AC during 1975 and 2016, which is probably associated with obesity, gastroesophageal reflux disease (GERD), Helicobacter pylori, and so on (13).

In our present study, we found that esophageal ASC is somewhat closer to AC rather than SqCC in terms of demographics and clinical characteristics. The mean age at diagnosis of ASC was 65.7, which was younger than AC (67.2) and SqCC (68.1). In the study of Evans et al., the median age was 65 for ASC, 65 for AC, and 67 for ASC. It seems that the age at diagnosis of ASC patients was younger, and was closer to AC than SqCC patients. Esophageal ASC is mainly found in the lower third or the abdominal portion of the esophagus resembling the distribution pattern of AC but tends to present a later summary stage or AJCC stage, a higher lymph node metastasis rate, and a higher distant metastasis than AC and SqCC. This is consistent with some published literature (14, 15). However, some studies showed that most esophageal ASC is present in the middle esophagus (6, 9, 12). Several studies suggested that Barrett's esophagus may be associated with ASC (16–18), and esophagitis caused by duodenal reflux has been shown to induce glandular metaplasia and ultimately, the occurrence of ASC in animal models (18). Perhaps it is because some of the occurrences of adenosquamous carcinoma are related to esophageal reflux that more than half of ASC appear in the lower third or the abdominal portion of the esophagus.

Current research is conflicting when it comes to the prognosis of ASC compared with AC and SqCC. In a large patient series conducted by Evans et al., the OS of ASC was lower than SqCC and AC (5). In contrast, Yachida et al. reported that the OS for ASC is better than AC and SCC (12), but this finding can be attributed to the smaller size. In Yendamuri et al.'s study, the OS of ASC seems equivalent to SqCC (4). Our study showed that the overall survival in patients with ASC is worse than that in patients with AC and SqCC. However, when stratified by gender, race, pathological grade, summary stage, AJCC stage, T stage, lymph node metastasis, M stage, and primary site, the prognosis of ASC seems closer to SqCC rather than AC. The OS between ASC and SqCC patients had a statistical difference only in the white race and pathological Grade NOS stratification variables. However, a statistical difference of OS between ASC and AC patients was found in the male, white race, Grade NOS, localized tumor stage, AJCC stage I, AJCC stage III, T1 stage, T4 stage, lymph node metastasis, and M0 stage stratification variables. The survival analyses of patients after PSM indicated that a poor prognosis of ASC may be associated with the tendency of ASC to present a later summary stage or AJCC stage, a higher lymph node metastasis rate, and a higher distant metastasis. We also found that gender, race, and lymph node metastasis have no influence on the OS of ASC patients which is different from AC and SqCC. The result that lymph node metastasis was irrelevant to the OS of ASC was unexpected and may be due to the different treatment strategies and the small number of ASC patients. In a study of gastric adenosquamous carcinoma, they also found that gender and ethnicity were not associated with OS (19). This suggested that gender and race were irrelevant to the prognosis of ASC.

To further explore the predictors of mortality in esophageal carcinoma, we conducted the univariate and multivariate Cox analysis and constructed a nomogram. In our study, histological type, age, gender, race, pathological grade, summary stage, AJCC stage, T stage, lymph node metastasis M stage, and primary site were regarded as independent prognostic factors for OS of all esophageal carcinoma patients as expected. Patients with the following factors: old age, male, black race, overlapping site, higher tumor grade, lymph node metastasis, higher summary stage and AJCC stage, may have a worse outcome, which is consistent with other studies (20, 21).

Besides, our study also had some limitations. Firstly, we excluded some patients for the absence of clinicopathologic data (such as AJCC stage, TNM stage, pathological grade) which could affect the accuracy of the results. Secondly, the SEER database had no detailed chemotherapy regimens available to assess the impacts of specific treatment regimens, so we did not extract radiation or chemotherapy information for our study. Thirdly, considering that the AJCC 7th edition was available since 2010, we just included patients from 2010–2016, the number of which is not large, especially the ASC patients. Finally, it is a retrospective study with an inherent bias which was performed from a database and the evaluation of the nomogram model was internal validation rather than external. Therefore, we built an online dynamic nomogram which we hope could be utilized and further validated in future clinical work. In upcoming studies, we will conduct prospective clinical trials in our center and verify our founding.

## Conclusion

Esophageal ASC is a rare type of esophageal cancer, only comprising <1% of all esophageal malignancies. However, ASC patients tended to present a poorer differentiation, later summary stage or AJCC stage, later T stage, higher lymph node metastasis rate, higher distant metastasis, and had a worse prognosis compared with AC or SqCC. Therefore, we should pay more attention to esophageal ASC, and future studies regarding the pathogenesis, biological behavior, treatment, and prognosis of ASC are required. Also, in our study, the histological type, age, gender, race, pathological grade, summary stage, AJCC stage, T stage, lymph node metastasis M stage, and primary site were regarded as independent prognostic factors for the OS of all esophageal carcinoma patients as expected.

## Data Availability Statement

The original contributions presented in the study are included in the article/Supplementary Material, further inquiries can be directed to the corresponding authors.

## Author Contributions

HQ designed the research and wrote the article. CL and XJ processed data and wrote the article. YD reviewed and edited the article. XL and GZ analyzed the data and reviewed the article. All authors contributed to the article and approved the submitted version.

## Conflict of Interest

The authors declare that the research was conducted in the absence of any commercial or financial relationships that could be construed as a potential conflict of interest.

## Supplementary Material

Supplementary Figure 1 Kaplan–Meier estimated overall survival in three types of patients stratified by gender **(A)**, race **(B)**, pathological grade **(C)**, summary stage **(D)**, AJCC stage **(E)**, T stage **(F)**, lymph node metastasis **(G)**, M stage **(H)**, primary site **(I)**.

Supplementary Figure 2 Kaplan–Meier estimated overall survival for propensity score weighted analyses of patients with adenosquamous carcinoma of the esophagus compared to those with adenocarcinoma **(A)** and squamous cell carcinoma **(B)** of the esophagus.

Supplementary Table 1 APC of each segment for esophageal ASC, AC, and SqCC.

Supplementary Table 2 Log-rank-test results of three cancer types in different stratification variable.
