# Supplementary figures and images for: Clinical Characteristics, Prognosis, and Nomogram for Esophageal Cancer Based on Adenosquamous Carcinoma: A SEER Database Analysis
Source: Front Oncol. 2021 Apr 26;11:603349. doi: 10.3389/fonc.2021.603349 (PMC8107687; doi:10.3389/fonc.2021.603349)

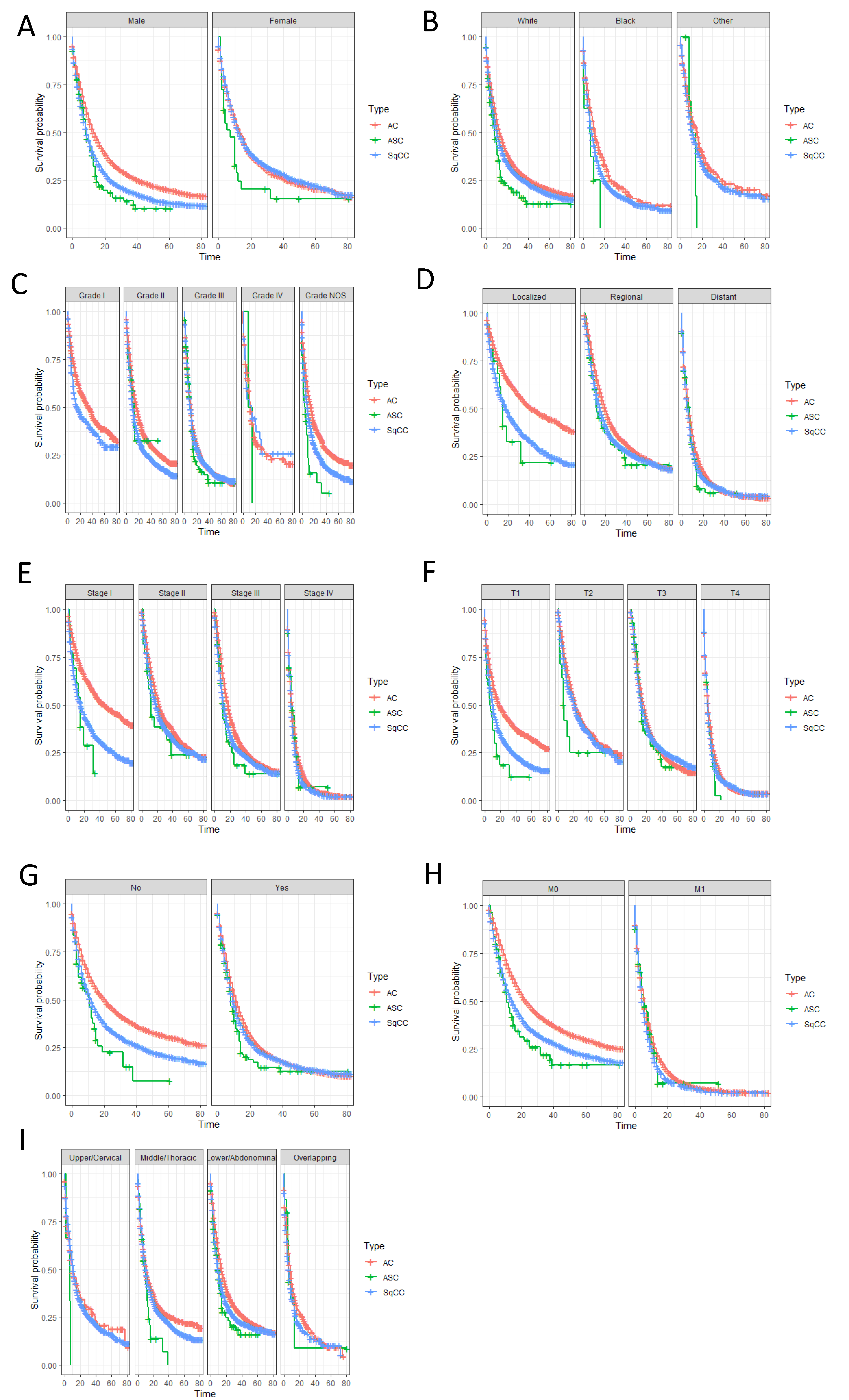

Supplement: Supplementary Figure 1 — Kaplan–Meier estimated overall survival in three types of patients stratified by gender (A), race (B), pathological grade (C), summary stage (D), AJCC stage (E), T stage (F), lymph node metastasis (G), M stage (H), primary site (I). [file Image_1.TIF]

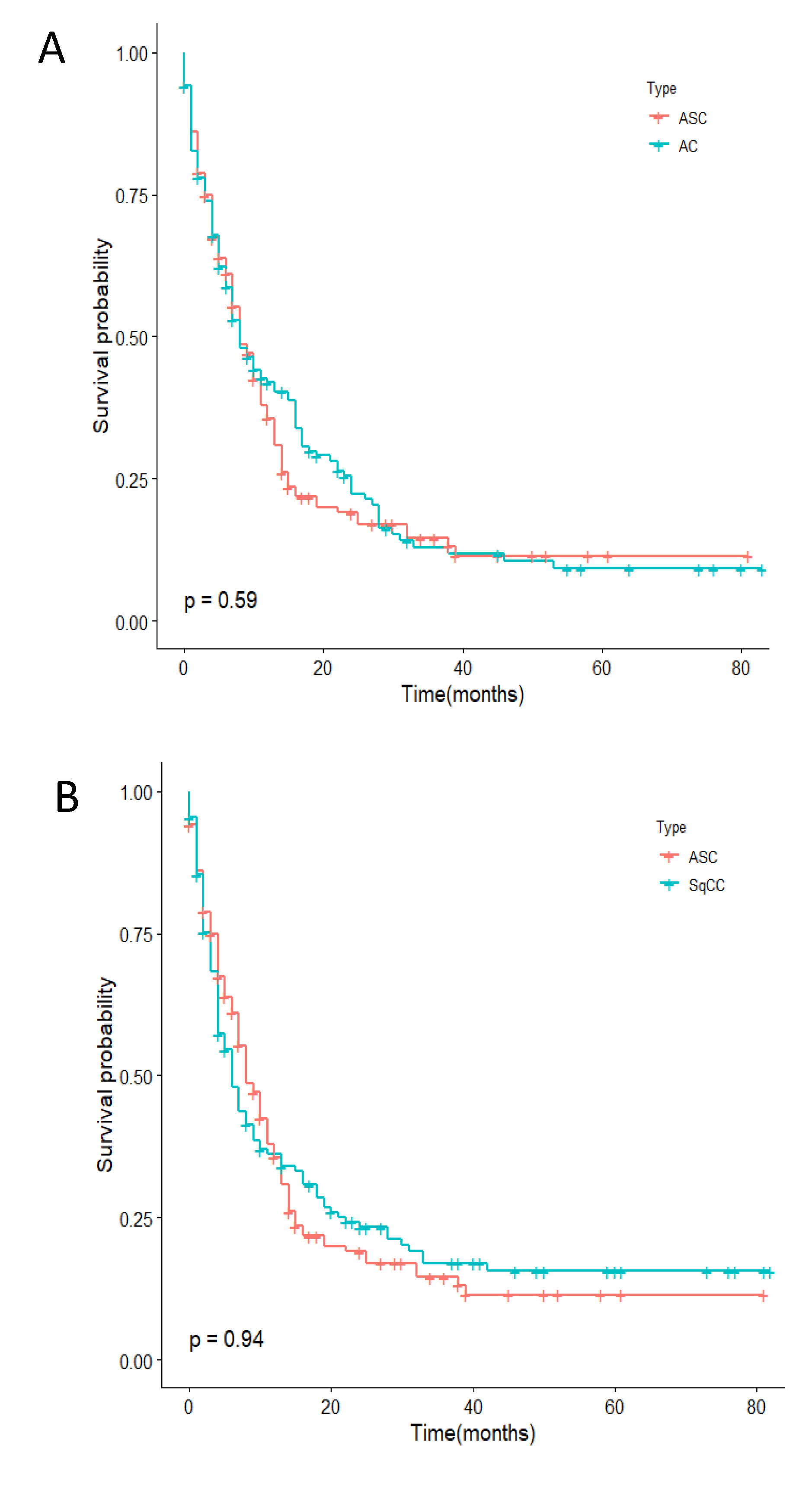

Supplement: Supplementary Figure 2 — Kaplan–Meier estimated overall survival for propensity score weighted analyses of patients with adenosquamous carcinoma of the esophagus compared to those with adenocarcinoma (A) and squamous cell carcinoma (B) of the esophagus. [file Image_2.TIFF]
