# Supplementary material for: Clinical Characteristics, Prognosis, and Nomogram for Esophageal Cancer Based on Adenosquamous Carcinoma: A SEER Database Analysis
Source: Front Oncol. 2021 Apr 26;11:603349. doi: 10.3389/fonc.2021.603349 (PMC8107687; doi:10.3389/fonc.2021.603349)
Supplement: Supplementary Table 1 — APC of each segment for esophageal ASC, AC, and SqCC. [file Table_1.docx]

**Supplementary Table 1. APC of each segment for esophageal ASC, AC and SqCC**

| Segment | Lower Endpoint | Upper Endpoint | APC | Lower CI | Upper CI |
| --- | --- | --- | --- | --- | --- |
| AC-1 | 1975 | 1999 | 7.5* | 6.9 | 8.2 |
| AC-2 | 1999 | 2016 | 0.6* | 0.1 | 1.2 |
| ASC-1 | 1975 | 1989 | 11.7* | 4.8 | 19 |
| ASC-2 | 1989 | 2016 | -3.1* | -4.6 | -1.5 |
| SqCC-1 | 1975 | 1986 | 0 | -0.7 | 0.7 |
| SqCC-2 | 1986 | 2012 | -3.2* | -3.4 | -3 |
| SqCC-3 | 2012 | 2016 | 0 | -3.7 | 3.7 |

AC: adenocarcinoma; SqCC: squamous cell carcinoma; ASC: adenosquamous carcinoma; APC: annual percent change. * indicated that the APC is significantly different from zero at the alpha = 0.05 level.
