# Supplementary material for: Clinical Characteristics, Prognosis, and Nomogram for Esophageal Cancer Based on Adenosquamous Carcinoma: A SEER Database Analysis
Source: Front Oncol. 2021 Apr 26;11:603349. doi: 10.3389/fonc.2021.603349 (PMC8107687; doi:10.3389/fonc.2021.603349)
Supplement: Supplementary Table 2 — Log-rank-test results of three cancer types in different stratification variable. [file Table_2.docx]

**Supplementary Table 2. Log-rank test results of three cancer types in different stratification variable**

| Stratification | ASC vs AC | ASC vs SqCC | AC vs SqCC |
| --- | --- | --- | --- |
| Gender |  |  |  |
| Male | <0.001 | 0.220 | <0.001 |
| Female | 0.058 | 0.058 | 0.600 |
| Race |  |  |  |
| White | <0.001 | *0.006 | <0.001 |
| Black | 0.067 | 0.162 | *0.02 |
| Other | 0.299 | 0.453 | 0.299 |
| Pathological grade |  |  |  |
| Grade I | —— | —— | <0.001 |
| Grade II | 0.700 | 0.700 | <0.001 |
| Grade III | 0.230 | 0.350 | 0.230 |
| Grade IV | 0.840 | 0.840 | 0.890 |
| Grade NOS | <0.001 | *0.007 | <0.001 |
| Summary stage |  |  |  |
| Localized | *0.024 | 0.604 | <0.001 |
| Regional | 0.159 | 0.670 | <0.001 |
| Distant | 0.094 | 0.490 | <0.001 |
| AJCC stage |  |  |  |
| Stage I | *0.004 | 0.414 | <0.001 |
| Stage II | 0.261 | 0.446 | *0.004 |
| Stage III | *0.032 | 0.529 | <0.001 |
| Stage IV | 0.522 | 0.610 | <0.001 |
| T stage |  |  |  |
| T1 | <0.001 | 0.082 | <0.001 |
| T2 | 0.092 | 0.092 | 0.092 |
| T3 | 0.702 | 0.702 | 0.702 |
| T4 | *0.044 | 0.157 | *0.032 |
| LNM |  |  |  |
| No | <0.001 | 0.073 | <0.001 |
| Yes | *0.017 | 0.115 | *0.001 |
| M stage |  |  |  |
| M0 | <0.001 | 0.176 | <0.001 |
| M1 | 0.522 | 0.610 | <0.001 |

AC: adenocarcinoma; SqCC: squamous cell carcinoma; ASC: adenosquamous carcinoma; LNM: lymph node metastasis; * indicated that the P-value is <0.005.
